# Curcumin Supplementation Reduces Inflammation, Neutrophil-to-Lymphocyte Ratio (NLR), and Antioxidant Status in Obese Patients with Type 2 Diabetes: A Randomized Controlled Trial

**DOI:** 10.3390/ijms27093854

**Published:** 2026-04-27

**Authors:** Metha Yaikwawong, Khanittha Kamdee, Somlak Chuengsamarn

**Affiliations:** 1 Department of Pharmacology, Faculty of Medicine Siriraj Hospital, Mahidol University, Bangkok 10700, Thailand; metha.yai@mahidol.ac.th (M.Y.); khanittha.kam@mahidol.ac.th (K.K.); 2 Division of Endocrinology and Metabolism, Faculty of Medicine, HRH Princess Maha Chakri Sirindhorn Medical Center, Srinakharinwirot University, Ongkharak, Nakhon Nayok 26120, Thailand

**Keywords:** type 2 diabetes mellitus, curcumin, anti-inflammatory effects, antioxidant effects, glycemic control, nutritional supplements

## Abstract

Type 2 diabetes mellitus (T2DM) is a chronic metabolic disorder characterized by insulin resistance and impaired insulin secretion, and curcumin—a polyphenolic compound derived from *Curcuma longa*—has shown potential anti-inflammatory and antioxidant effects. This randomized, double-blind, placebo-controlled trial evaluated the effects of 1500 mg/day curcumin supplementation for 12 months in 114 adults with T2DM, with assessments including fasting plasma glucose (FPG), glycated hemoglobin (HbA1c), insulin resistance (HOMA-IR), inflammatory cytokines (IL-6, IL-1β, TNF-α), high-sensitivity C-reactive protein (hs-CRP), neutrophil-to-lymphocyte ratio (NLR), antioxidant markers (SOD, GPx, TAS), and malondialdehyde (MDA). Curcumin supplementation was associated with significant reductions in pro-inflammatory cytokines (*p* < 0.001), hs-CRP and NLR (*p* < 0.05), and with improved antioxidant status as shown by increased TAS, SOD, and GPx together with reduced MDA levels (*p* < 0.001). Additionally, improvements in metabolic parameters were observed, including lower FPG (112.0 mg/dL vs. 134.5 mg/dL; *p* < 0.001), HbA1c (6.10% vs. 6.40%; *p* < 0.05), and HOMA-IR (4.88 vs. 6.71; *p* < 0.001). Overall, the findings suggest that long-term curcumin supplementation may contribute to improved inflammatory, antioxidant, and glycemic profiles in obese individuals with T2DM; however, further multi-center studies are needed to confirm these observations and clarify their clinical relevance.

## 1. Introduction

Type 2 diabetes mellitus (T2DM) has become a significant global health concern, with its prevalence increasing markedly over recent decades. According to the International Diabetes Federation (IDF) Diabetes Atlas, approximately 10.5% of adults aged 20 to 79 years were living with diabetes in 2021, equating to 536.6 million individuals. Projections indicate that this figure could rise to 12.2% by 2045, affecting an estimated 783.2 million people [1]. Recent data from the World Health Organization (WHO) indicates that the global prevalence of diabetes among adults has more than doubled, increasing from 6.8% in 1990 to 14.1% in 2022. This significant rise underscores the escalating global health challenge posed by diabetes [2]. This upward trend underscores the urgent need for effective prevention and management strategies to address the escalating burden of T2DM worldwide. T2DM and obesity are interrelated conditions that significantly elevate the risk of cardiovascular diseases and other health complications [3]. Beyond the substantial human toll, the economic burden of T2DM is immense. In 2021, global diabetes-related health expenditure was estimated at USD 966 billion, representing a 316% increase over the past 15 years, with costs projected to exceed USD 1.05 trillion by 2045 [4]. These escalating financial costs, driven by direct medical expenses and indirect productivity losses, place a substantial strain on healthcare systems and economies worldwide. This upward trend underscores the urgent need for effective prevention and management strategies to address the escalating burden of T2DM globally [5].

Chronic low-grade inflammation and oxidative stress are pivotal in the development of T2DM and obesity. Inflammation contributes to insulin resistance and metabolic dysregulation, exacerbating the progression of metabolic syndrome (MetS) and T2DM [6]. Oxidative stress disrupts insulin signaling and impairs β-cell function, further compromising glucose metabolism. The interplay between these processes underscores the importance of targeting both inflammation and oxidative stress in therapeutic strategies for T2DM and obesity [6,7]. Current antidiabetic medications exhibit anti-inflammatory and antioxidant properties, contributing to weight loss and reduced cardiometabolic risks in T2DM patients [8]. Glucagon-Like Peptide-1 Receptor Agonists (GLP-1 RAs) such as semaglutide and liraglutide not only enhance glycemic control but also promote significant weight loss and reduce cardiovascular events. These benefits are partly attributed to their anti-inflammatory and antioxidant effects, which improve metabolic and cardiovascular outcomes [8,9]. Dipeptidyl peptidase-4 (DPP-4) inhibitors like sitagliptin and linagliptin have demonstrated anti-inflammatory benefits. Research indicates that sitagliptin therapy can lead to significant decreases in C-reactive protein (CRP) levels, suggesting a reduction in systemic inflammation among T2DM patients [10]. Linagliptin has been shown to reduce obesity-related inflammation and insulin resistance [8,11]. Sodium-Glucose Cotransporter-2 (SGLT-2) Inhibitors such as empagliflozin and dapagliflozin exhibit anti-inflammatory and antioxidant properties, contributing to cardioprotective effects. These agents reduce markers of oxidative stress and inflammation, potentially offering protective benefits against diabetic complications and improving cardiovascular outcomes [8,12]. The high cost of conventional medications has led to growing interest in alternative therapeutic approaches, particularly the use of nutritional supplements and medicinal plants, as potential cost-effective and accessible options for managing health conditions.

Nutraceuticals present a viable alternative for low-risk patients, serving either as an adjunct to current therapies or as an initial treatment option prior to the introduction of pharmaceutical interventions in non-high-risk cases [13]. Several herbal supplements have shown promising antioxidant and anti-inflammatory properties, which may provide therapeutic benefits for managing T2DM. These include berberine, an alkaloid derived from *Coptis chinensis* [14]; cinnamon (*Cinnamomum cassia*) [15]; and curcumin, the active component of turmeric (*Curcuma longa*) [16].

Curcumin is a well-known member of the diarylheptanoid family, a class of natural products defined by two aromatic rings connected by a seven-carbon aliphatic chain. This scaffold has gained considerable attention due to its biological relevance, particularly its potent anti-inflammatory and antioxidant activities [17]. Derived from turmeric (*Curcuma longa*), curcumin has been extensively studied for its ability to modulate inflammatory and oxidative pathways [18,19]. However, curcumin is characterized by inherently poor systemic bioavailability due to rapid hepatic metabolism, extensive glucuronidation, and limited intestinal absorption [20]. Despite this pharmacokinetic limitation, preclinical evidence indicates that curcumin improves insulin sensitivity and glycemic control by downregulating key pro-inflammatory cytokines, including interleukin-6 (IL-6), interleukin-1 beta (IL-1β), and tumor necrosis factor-alpha (TNF-α) [21,22,23].

These findings are supported by clinical studies showing that curcumin supplementation reduces inflammatory and oxidative stress markers in patients with type 2 diabetes mellitus (T2DM) and obesity. In our previous work, curcumin intervention in prediabetic and diabetic individuals was shown to reduce the progression from prediabetes to T2DM, enhance β-cell function, and decrease insulin resistance (IR) [24,25]. Recent systematic reviews and meta-analyses have further consolidated this evidence, confirming that curcumin supplementation significantly attenuates hyperglycemia and reduces circulating levels of pro-inflammatory cytokines and oxidative stress markers in T2DM populations [26]. Additionally, umbrella reviews have highlighted the consistency of curcumin’s beneficial effects on metabolic parameters across multiple randomized controlled trials [27].

Building on these findings, we extended the study duration from 6 to 12 months to evaluate the long-term effects of curcumin, particularly in patients with obesity and T2DM. Additionally, we assessed the effects of curcumin supplementation on levels of pro-inflammatory cytokines, such as interleukin-6 (IL-6), interleukin 1-beta (IL-1β), tumor necrosis factor alpha (TNF-α), oxidative stress markers, and adipokine-related indicators in a large, randomized, double-blind, placebo-controlled study.

## 2. Results

**Figure 1:**
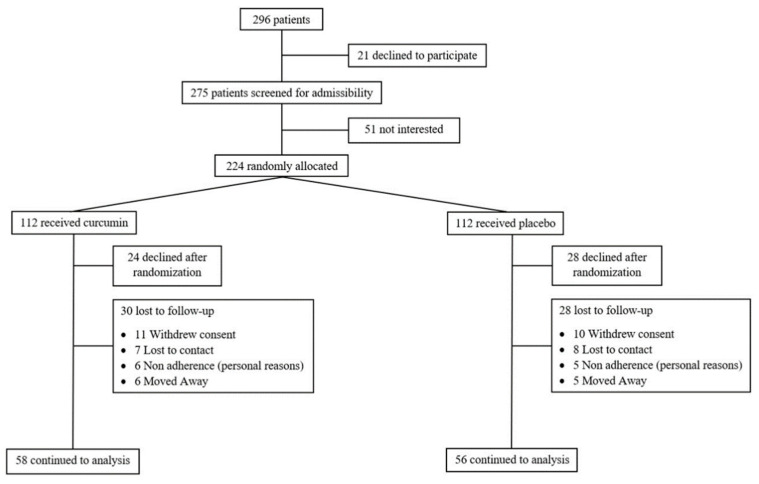
CONSORT flow diagram of participant enrollment and allocation.

A flow chart of the trial is provided in Figure 1. Initially, 296 subjects were enrolled in the study. Baseline characteristics of the 114 subjects randomly assigned to the two groups are detailed in Table 1. No statistically significant differences were observed in any baseline parameters between the placebo-treated group and the curcumin-treated group.

### 2.1. Intervention Outcomes

The intervention outcomes were evaluated across several domains, encompassing anti-inflammatory, antioxidant effects, metabolic profile effects, glycemic control effects, and the assessment of potential adverse effects.

#### 2.1.1. Anti-Inflammatory Outcome

As presented in Table 2, there were no significant differences between the placebo and curcumin groups at baseline for inflammatory markers, including IL-6, IL-1β, TNF-α, neutrophil-to-lymphocyte ratio (NLR), and hs-CRP (all NS), indicating good baseline comparability.

**Figure 2:**
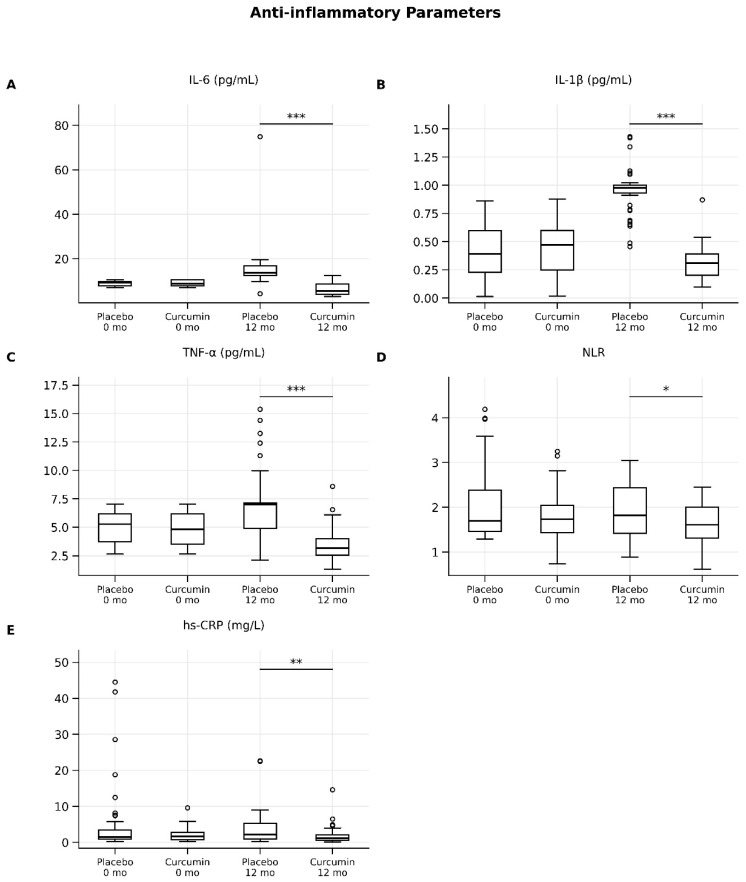
Box-and-whisker plots show changes in circulating inflammatory markers in the placebo and curcumin groups at baseline (0 months) and after 12 months of intervention. (**A**) Interleukin-6 (IL-6), (**B**) interleukin-1 beta (IL-1β), (**C**) tumor necrosis factor-alpha (TNF-α), (**D**) neutrophil-to-lymphocyte ratio (NLR), and (**E**) high-sensitivity C-reactive protein (hs-CRP). Data are presented as medians with interquartile ranges (IQRs); whiskers indicate minimum and maximum values, and circles represent outliers. Between-group comparisons at 12 months were performed using the Mann–Whitney U test. * *p* < 0.05; ** *p* < 0.01; *** *p* < 0.001.

At 12 months, curcumin supplementation significantly reduced pro-inflammatory cytokines compared with placebo (Table 2; Figure 2). Median IL-6 levels were markedly lower in the curcumin group than in the placebo group (5.50 [4.39–9.00] vs. 13.69 [11.27–15.65], *p* < 0.001), with a large effect size (*r* = 0.78, 95% CI: 0.68–0.86). Similarly, IL-1β (0.31 [0.20–0.39] vs. 0.98 [0.94–1.01], *p* < 0.001; *r* = 0.86, 95% CI: 0.78–0.92) and TNF-α (3.17 [2.45–3.90] vs. 7.00 [5.89–8.10], *p* < 0.001; *r* = 0.84, 95% CI: 0.75–0.90) were significantly reduced, both demonstrating large effect sizes. In addition, hs-CRP levels were significantly lower in the curcumin group compared to placebo (1.17 [0.57–2.06] vs. 2.22 [1.08–5.37], *p* = 0.001), corresponding to a moderate-to-large effect size (*r* = 0.59, 95% CI: 0.46–0.70). The reduction in NLR was smaller but remained statistically significant (1.61 [1.23–1.92] vs. 1.82 [1.31–2.34], *p* = 0.029), with a small effect size (*r* = 0.27, 95% CI: 0.12–0.41).

Overall, these results demonstrate that curcumin supplementation exerts significant anti-inflammatory effects, particularly in reducing circulating cytokines, with consistently large effect sizes observed for IL-6, IL-1β, and TNF-α (Table 2; Figure 2).

#### 2.1.2. Effects of Curcumin on Neutrophil-to-Lymphocyte Ratio: Sex-Stratified and Sensitivity Analyses

Sex-stratified analyses of changes in the neutrophil-to-lymphocyte ratio (NLR) are presented in Table 3. In the placebo group, both males and females exhibited slight reductions in NLR over the study period (mean change: −0.124 ± 0.822 in males and −0.030 ± 0.723 in females). In contrast, participants receiving curcumin demonstrated greater changes, with mean increases of 0.537 ± 0.940 in males and 0.276 ± 0.693 in females. However, despite positive mean values in some subgroups, median-based and regression analyses consistently indicated an overall reduction in NLR following curcumin supplementation. Although the magnitude of change appeared numerically greater in males than in females, this difference was not statistically evaluated for significance.

To assess the robustness of the treatment effect, multiple sensitivity analyses were conducted (Table 4). In the primary linear regression model, curcumin treatment was associated with a significant between-group difference in NLR change (β = 0.484, 95% CI: 0.227–0.741, *p* < 0.001). This finding remained consistent across alternative analytical approaches, including robust regression using Huber–White standard errors (β = 0.484, 95% CI: 0.233–0.735, *p* < 0.001), quantile regression at the median (β = 0.523, 95% CI: 0.244–0.802, *p* < 0.001), and a non-parametric permutation test (*p* = 0.001), indicating that the observed effect was not sensitive to model assumptions.

The potential modifying effect of sex was further evaluated by including a treatment-by-sex interaction term in the regression model (Table 5). In the primary linear regression model, curcumin treatment remained significantly associated with NLR change (β = 0.661, 95% CI: 0.147–1.175, *p* = 0.012), whereas sex was not a significant predictor (*p* = 0.717). Importantly, the interaction between treatment and sex was not statistically significant (β = −0.344, 95% CI: −1.069 to 0.382, *p* = 0.352), suggesting that the effect of curcumin on NLR change was consistent across males and females. These findings were corroborated by robust regression, quantile regression, and permutation-based analyses, all of which demonstrated similar effect estimates and confirmed the absence of a significant interaction (all *p* > 0.05).

Overall, these results indicate that curcumin supplementation significantly influences NLR, with consistent effects across multiple statistical approaches and no evidence of sex-specific modification.

#### 2.1.3. Antioxidant Defense Outcome

**Figure 3:**
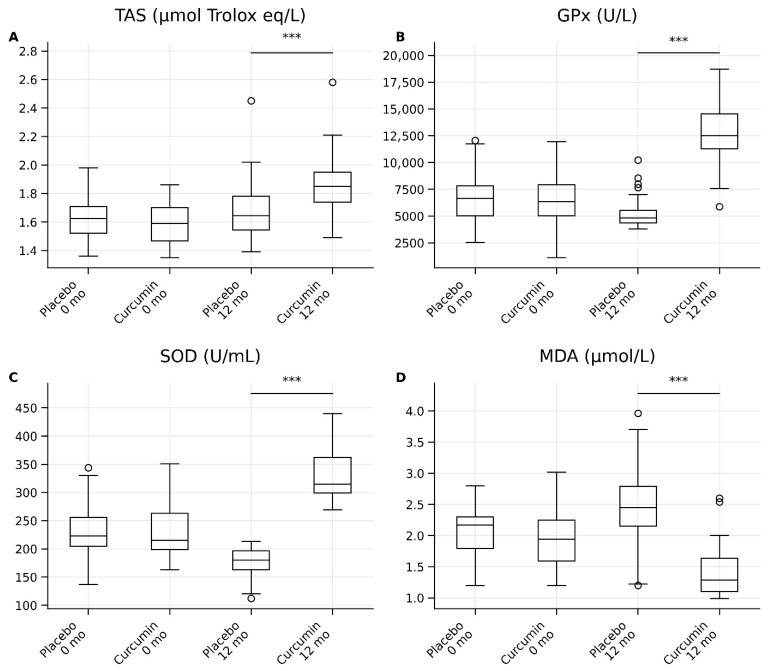
Box-and-whisker plots show antioxidant and oxidative stress parameters in the placebo and curcumin groups at baseline (0 months) and after 12 months of intervention. (**A**) Total antioxidant status (TAS), (**B**) glutathione peroxidase (GPx), (**C**) superoxide dismutase (SOD), and (**D**) malondialdehyde (MDA). Data are presented as medians with interquartile ranges (IQRs); whiskers indicate minimum and maximum values, and circles represent outliers. Between-group comparisons at 12 months were performed using the Mann–Whitney U test. *** *p* < 0.001.

As shown in Table 2 and Figure 3, there were no significant differences between the placebo and curcumin groups at baseline for antioxidant markers, including total antioxidant status (TAS), glutathione peroxidase (GPx), superoxide dismutase (SOD), and malondialdehyde (MDA) (all *p* > 0.05), indicating comparable baseline oxidative status.

At 12 months, curcumin supplementation resulted in significant improvements in antioxidant capacity compared with placebo (Table 2; Figure 3). TAS levels were significantly higher in the curcumin group than in the placebo group (1.85 [1.74–1.95] vs. 1.65 [1.55–1.79], *p* < 0.001), with a large effect size (*r* = 0.72, 95% CI: 0.61–0.81). Similarly, GPx activity was markedly increased in the curcumin group (12,533.0 [11,234.8–14,562.5] vs. 4820.0 [4448.8–5611.3], *p* < 0.001), demonstrating a large effect size (*r* = 0.89, 95% CI: 0.83–0.93). SOD levels were also significantly elevated following curcumin supplementation (315 [284–347] vs. 180 [169–203], *p* < 0.001), with a large effect size (*r* = 0.87, 95% CI: 0.80–0.92). Conversely, MDA levels, a marker of lipid peroxidation, were significantly lower in the curcumin group compared with placebo (1.29 [1.05–1.58] vs. 2.45 [2.14–2.77], *p* < 0.001), corresponding to a large effect size (*r* = 0.85, 95% CI: 0.77–0.91).

Overall, these findings demonstrate that long-term curcumin supplementation significantly enhances antioxidant defense systems while reducing oxidative stress, with consistently large effect sizes observed across all antioxidant markers (Table 2; Figure 3).

#### 2.1.4. Glycemic Control Outcomes

**Figure 4:**
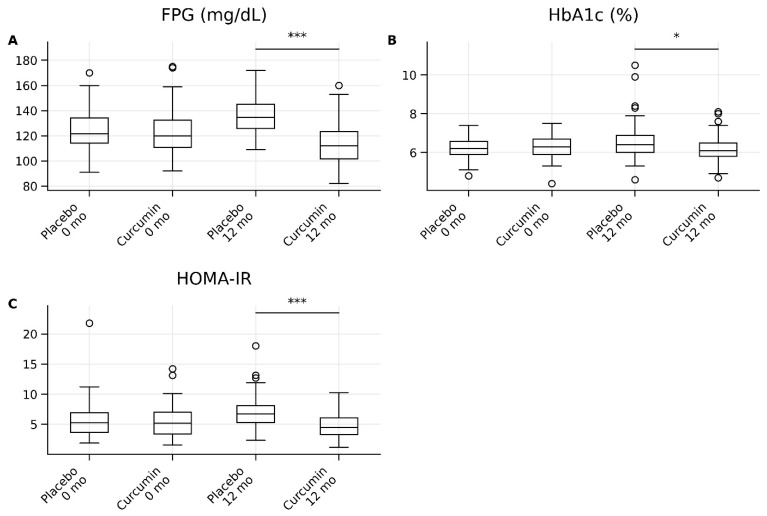
Box-and-whisker plots depict glycemic parameters in the placebo and curcumin groups at baseline (0 months) and after 12 months of intervention. (**A**) Fasting plasma glucose (FPG), (**B**) glycated hemoglobin (HbA1c), and (**C**) insulin resistance assessed by the homeostatic model assessment of insulin resistance (HOMA-IR). Data are presented as medians with interquartile ranges (IQRs); whiskers indicate minimum and maximum values, and circles represent outliers. Between-group comparisons at 12 months were performed using the Mann–Whitney U test. * *p* < 0.05; *** *p* < 0.001.

As shown in Table 6 and Figure 4, there were no significant differences between the placebo and curcumin groups at baseline for fasting plasma glucose (FPG), glycated hemoglobin (HbA1c), and insulin resistance as assessed by HOMA-IR, indicating comparable glycemic status between groups.

At 12 months, curcumin supplementation resulted in significant improvements in glycemic parameters compared with placebo (Table 6; Figure 4). FPG levels were significantly lower in the curcumin group than in the placebo group (112.0 [101.3–122.8] vs. 134.5 [125.3–144.5], *p* < 0.001), with a large effect size (*r* = 0.71, 95% CI: 0.60–0.80). Similarly, HOMA-IR was significantly reduced in the curcumin group (4.88 [3.49–6.28] vs. 6.71 [5.31–8.11], *p* < 0.001), also demonstrating a large effect size (*r* = 0.69, 95% CI: 0.58–0.78).

HbA1c levels were modestly but significantly lower in the curcumin group compared with placebo at 12 months (6.10 [5.80–6.50] vs. 6.40 [6.03–6.91], *p* = 0.019), corresponding to a moderate-to-large effect size (*r* = 0.52, 95% CI: 0.38–0.64).

Overall, these findings indicate that long-term curcumin supplementation significantly improves glycemic control and insulin resistance, with large effect sizes observed for FPG and HOMA-IR and a moderate-to-large effect for HbA1c (Table 6; Figure 4).

#### 2.1.5. Anthropometric Control Outcomes

At baseline, there were no significant differences between the placebo and curcumin groups in BMI or waist circumference (*p* > 0.05 for all; Table 7). After 12 months of intervention, participants in the placebo group exhibited a modest increase in both BMI and waist circumference, whereas those receiving curcumin supplementation demonstrated significantly lower values for both anthropometric measures.

Specifically, BMI at 12 months was significantly lower in the curcumin group compared with the placebo group (25.97 [24.22–27.78] vs. 26.57 [24.56–29.00] kg/m^2^; *p* = 0.036), with a moderate effect size (*r* = 0.42, 95% CI: 0.28–0.55). In addition, waist circumference was significantly reduced in the curcumin group compared with placebo at follow-up (88.0 [84–93] vs. 94 [90–98] cm; *p* = 0.001), corresponding to a large effect size (*r* = 0.61, 95% CI: 0.48–0.72) (Table 7).

**Figure 5:**
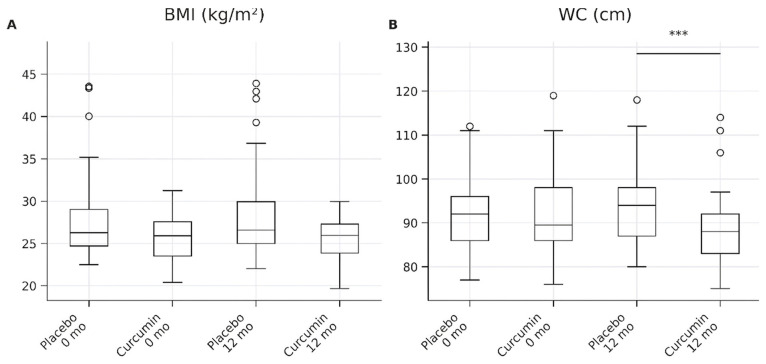
Box-and-whisker plots show anthropometric parameters in the placebo and curcumin groups at baseline (0 months) and after 12 months of intervention. (**A**) Body mass index (BMI) and (**B**) waist circumference (WC). Data are presented as medians with interquartile ranges (IQRs); whiskers indicate minimum and maximum values, and circles represent outliers. Between-group comparisons at 12 months were performed using the Mann–Whitney U test. *** *p* < 0.001.

These between-group differences are further illustrated in Figure 5, which demonstrates a clear attenuation of increases in BMI and waist circumference over time in the curcumin group compared with the progressive increases observed in the placebo group.

#### 2.1.6. Analysis of Changes from Baseline

To assess the net effect of the intervention, the change from baseline to 12 months (Δ = 12-month value − baseline value) was calculated for each outcome and compared between the curcumin and placebo groups. As shown in Table 8, the median change in all inflammatory, antioxidant, and glycemic parameters was significantly greater in the curcumin group compared to the placebo group. For instance, the reduction in IL-6 was significantly larger in the curcumin group than in the placebo group (median Δ: −3.30 pg/mL vs. +4.45 pg/mL; *p* < 0.001). Similarly, the improvement in total antioxidant status was significantly greater with curcumin (median Δ: +0.26 μmol trolox eq/L) compared to placebo (median Δ: +0.03 μmol trolox eq/L; *p* < 0.001). These change-score analyses confirm that the differences observed at 12 months are attributable to the intervention rather than to any minor baseline imbalances.

#### 2.1.7. Adverse Effects

All reported adverse effects were graded according to the Common Terminology Criteria for Adverse Events (CTCAE v5.0). The adverse events observed in the curcumin group—abdominal discomfort (10.7%), diarrhea (5.4%), and headache (3.6%)—were classified as Grade 1 (mild) and self-limiting. No Grade 2–4 events were identified. Investigators assessed causality based on temporal association, recurrence upon continued dosing, and absence of alternative explanations. Abdominal discomfort and diarrhea were judged as possibly related to curcumin supplementation due to known gastrointestinal sensitivity to herbal compounds, whereas headaches were classified as unlikely to be related. No serious adverse events (SAEs) occurred in either group (Table 9), and no participants required dose reductions, temporary treatment interruptions, or study withdrawal due to side effects. However, the study was not powered to detect rare adverse events, and the long-term safety profile beyond 12 months remains to be established. All participants tolerated the full prescribed regimen of 1500 mg/day of curcumin or a matching placebo for the entire 12-month period. Routine monitoring of hepatic and renal function revealed no clinically meaningful changes, and mean levels of AST, ALT, and creatinine remained comparable between groups at both baseline and 12 months (Table 10).

#### 2.1.8. Sensitivity Analyses and Consistency Between Per-Protocol and Intention-to-Treat Analyses

Sensitivity analyses demonstrated that the effects of curcumin supplementation were consistent across different analytical approaches (Table 11 and Table 12). For the primary outcome, changes in neutrophil-to-lymphocyte ratio (NLR) remained statistically significant across all methods, including per-protocol, intention-to-treat (ITT) with multiple imputation, complete case analysis, and last observation carried forward (LOCF). The estimated treatment effect was comparable across models, ranging from −0.138 to −0.176, with all confidence intervals excluding the null and *p*-values < 0.05, indicating robustness of the findings to different assumptions regarding missing data (Table 11).

Similarly, comparisons between per-protocol and ITT analyses for both primary and secondary outcomes showed highly consistent results (Table 12). Curcumin supplementation was associated with significant reductions in anthropometric measures, including BMI and waist circumference, with comparable effect sizes between analytical approaches. Inflammatory markers, including NLR, hs-CRP, IL-6, IL-1β, and TNF-α, were significantly reduced in both per-protocol and ITT analyses, with closely aligned effect estimates and overlapping confidence intervals.

Consistent improvements were also observed in glycemic control, with significant reductions in HbA1c, fasting plasma glucose (FPG), and HOMA-IR across both analytical frameworks. In addition, antioxidant markers demonstrated robust and concordant effects, with significant increases in total antioxidant status (TAS), glutathione peroxidase (GPx), and superoxide dismutase (SOD), alongside reductions in malondialdehyde (MDA), in both per-protocol and ITT analyses.

Overall, the consistency of effect estimates across multiple analytical strategies supports the robustness and reliability of the observed benefits of curcumin supplementation, indicating that the findings are not sensitive to the method of analysis or handling of missing data.

## 3. Discussion

This randomized, double-blind, placebo-controlled trial demonstrates that 12 months of curcumin supplementation exerts significant and predominantly large anti-inflammatory and antioxidant effects across multiple biomarkers in patients with type 2 diabetes mellitus (T2DM) and obesity, accompanied by improvements in insulin resistance, glycemic control, and anthropometric parameters, including body mass index (BMI) and waist circumference. These findings support curcumin as a potential adjunctive therapy and address a key gap in the literature regarding long-term intervention and comprehensive biomarker assessment.

### 3.1. Anti-Inflammatory Effects

Curcumin supplementation significantly reduced circulating pro-inflammatory cytokines (IL-6, IL-1β, TNF-α) and high-sensitivity C-reactive protein (hs-CRP), consistent with prior shorter-term studies in T2DM and obesity [28,29]. In parallel, curcumin significantly reduced the neutrophil-to-lymphocyte ratio (NLR), a marker of systemic inflammation associated with diabetic complications [30,31]. This effect remained consistent across sex-stratified analyses. These effects were supported by large effect sizes for IL-6, IL-1β, and TNF-α, and a moderate-to-large effect for hs-CRP, whereas the effect on NLR was small. However, the reduction in NLR (median 1.61 vs. 1.82; *p* = 0.029) should be interpreted cautiously. NLR is sensitive to transient factors such as infection and concomitant medications [32,33], which were not systematically assessed. Additionally, the modest between-group difference and overlapping interquartile ranges suggest limited clinical impact. Thus, NLR should be considered a supportive rather than definitive indicator of anti-inflammatory efficacy.

### 3.2. Antioxidant Effects

Curcumin demonstrated significant antioxidant effects, evidenced by increased superoxide dismutase (SOD), glutathione peroxidase (GPx), and total antioxidant status (TAS), alongside reduced malondialdehyde (MDA). These findings are consistent with previous reports [34,35] and are biologically plausible. Curcumin exerts direct free radical scavenging activity and activates the Nrf2 signaling pathway, leading to upregulation of endogenous antioxidant enzymes [36,37]. Given the central role of oxidative stress in insulin resistance and β-cell dysfunction in T2DM [6], these effects likely contribute to the observed metabolic improvements.

### 3.3. Glycemic Control, Insulin Sensitivity, and Anthropometric Changes

Curcumin supplementation resulted in significant reductions in HOMA-IR, fasting plasma glucose (FPG), and HbA1c, consistent with previous clinical studies [38,39]. Notably, these metabolic improvements were accompanied by modest but significant reductions in BMI and waist circumference, suggesting a beneficial effect on overall and central adiposity. These anthropometric changes are biologically plausible and may be mediated through curcumin’s anti-inflammatory effects in adipose tissue, modulation of adipokine secretion, and potential influences on lipid metabolism and energy homeostasis [40,41]. Given the central role of visceral adiposity in insulin resistance and chronic low-grade inflammation [42], reductions in waist circumference may have contributed to the observed improvements in glycemic and inflammatory markers. The convergence of reduced inflammation, enhanced antioxidant capacity, improved glycemic control, and decreased adiposity supports a multifactorial mechanism involving interconnected metabolic pathways. The extended 12-month duration of this study may have been critical in allowing these cumulative effects to become detectable.

### 3.4. Clinical Significance of Key Findings

Beyond statistical significance, the magnitude of change in several biomarkers is clinically relevant. The 0.30% reduction in HbA1c meets the minimum threshold for clinical meaningfulness (≥0.3–0.5%) defined by the American Diabetes Association, while the 22.5 mg/dL reduction in FPG brought mean values within recommended targets (<130 mg/dL) [43].

The 1.05 mg/L reduction in hs-CRP is also notable, as reductions of ≥0.5–1.0 mg/L are associated with meaningful cardiovascular risk reduction [44]. In addition, the observed reductions in BMI and waist circumference, although modest, further support potential cardiometabolic benefit, particularly given the established link between central obesity and cardiovascular risk [45].

The consistency of improvements across inflammatory, antioxidant, glycemic, and anthropometric domains strengthens the clinical relevance of the findings and suggests potential implications for long-term vascular outcomes. Importantly, these findings were highly consistent across multiple analytical approaches, including intention-to-treat, per-protocol, and sensitivity analyses, reinforcing the robustness and reliability of the observed effects.

### 3.5. Translational Considerations

While the observed improvements in HbA1c, hs-CRP, and anthropometric measures meet clinically meaningful thresholds, their magnitude remains modest compared with standard pharmacotherapies. Although statistically robust and supported by large effect sizes, the absolute clinical improvements remain modest compared with standard pharmacotherapies. For example, metformin typically reduces HbA1c by 1.0–1.5%, and SGLT2 inhibitors by 0.5–1.0% [46]. Accordingly, curcumin should be regarded as an adjunct rather than an alternative to established therapies.

A key translational challenge is the variability in commercially available curcumin formulations. The present study used a standardized extract (1500 mg/day) without bioavailability enhancers, whereas many products include agents such as piperine or employ nano- or phospholipid-based delivery systems [47]. These differences can substantially affect systemic exposure and clinical efficacy. Therefore, the present findings may not be directly generalizable to all formulations, particularly those with unverified composition or quality.

Clinical application should also consider patient selection. The benefits observed here were limited to individuals with recently diagnosed, well-controlled T2DM and may not extend to patients with more advanced disease. If used, curcumin should complement standard care, with appropriate monitoring for potential adverse effects and drug interactions. Overall, while curcumin represents a reasonable adjunctive option in selected patients, variability in formulation and modest effect size necessitate cautious interpretation.

### 3.6. Bioavailability Considerations and Mechanistic Plausibility

Despite the well-documented low systemic bioavailability of curcumin, the observed effects are biologically plausible [20]. Plasma concentrations alone may not reflect biological activity, as curcumin undergoes tissue accumulation and enterohepatic recirculation [48].

Gut-mediated mechanisms may also contribute, as curcumin can modulate the gut microbiome and reduce metabolic endotoxemia [49]. In addition, curcumin metabolites retain biological activity and may contribute to systemic effects [50]. The extended duration of supplementation likely enabled cumulative activation of antioxidant pathways (e.g., Nrf2) and gradual attenuation of chronic inflammation [51]. However, these findings may not be generalizable to shorter interventions or different formulations.

### 3.7. Strengths and Limitations

This study has several strengths, including its 12-month duration, randomized double-blind placebo-controlled design, and comprehensive assessment of inflammatory, oxidative, metabolic, and anthropometric outcomes. However, several limitations should be acknowledged. First, the strict inclusion criteria and single-center setting may limit the generalizability of the findings, while the relatively modest sample size may reduce statistical power. Second, the single-dose design precludes evaluation of dose–response relationships. Third, the absence of bioavailability enhancers and the lack of pharmacokinetic measurements limit comparisons with enhanced curcumin formulations and may affect the interpretation of systemic exposure–response relationships. Fourth, although pharmacological confounding was minimized through stable medication use, adherence to diet and lifestyle was not objectively quantified; therefore, unmeasured behavioral changes may have influenced outcomes sensitive to short-term variation, particularly inflammatory markers such as cytokines and the neutrophil-to-lymphocyte ratio (NLR). In addition, the incomplete capture of acute inflammatory events or interim medication changes may have introduced variability in these biomarkers. Finally, although no major safety concerns were observed, the study was not powered to detect rare adverse events, and longer-term safety remains uncertain. Future studies should incorporate larger, multicenter designs with pharmacokinetic assessments and more rigorous monitoring of adherence, acute clinical events, and concomitant therapies to strengthen causal inference.

## 4. Methods

### 4.1. Study Design and Participants

This study was designed as a randomized, double-blind, placebo-controlled clinical trial conducted at Princess Maha Chakri Sirindhorn Medical Center, Srinakharinwirot University, Nakhon Nayok, Thailand. The trial was conducted and reported in accordance with the Consolidated Standards of Reporting Trials (CONSORT) guidelines (Supplementary Figure S1). A total of 296 individuals with type 2 diabetes mellitus (T2DM) were screened for eligibility based on predefined inclusion and exclusion criteria. The overall study design, including participant enrollment, run-in period, randomization, intervention, and outcome assessment, is illustrated in the study flowchart presented in Supplementary Figure S2.

The study protocol consisted of a 3-month run-in period (months −3 to 0), followed by a 12-month intervention phase. During the run-in period, all participants received standardized education addressing dietary modification and physical activity. Upon entering the intervention phase, participants were provided with written lifestyle recommendations and participated in individualized counseling sessions lasting approximately 20–30 min, focusing on healthy lifestyle behaviors. Medical nutrition therapy and regular physical activity were emphasized throughout the study period.

All participants received metformin as monotherapy for glycemic management, and the use of additional antidiabetic medications was not permitted during recruitment to reduce potential confounding effects. Concomitant treatments for hypertension and dyslipidemia were managed according to standard clinical practice and remained unchanged throughout the trial (Table 1). No modifications were made to glucose-lowering or cardiometabolic treatment regimens during the intervention.

Eligible participants were adults aged ≥35 years with a diagnosis of T2DM within the previous year. Inclusion criteria required stable glycemic control at screening, defined as hemoglobin A1c (HbA1c) < 6.5% and fasting plasma glucose (FPG) < 120 mg/dL, as well as a body mass index (BMI) ≥ 23 kg/m^2^. These criteria were selected to ensure enrollment of individuals with early-stage, well-controlled T2DM, consistent with the study aim to examine outcomes prior to significant disease progression. T2DM was diagnosed in accordance with the 2017 American Diabetes Association criteria [52], which include an FPG ≥ 126 mg/dL, a 2 h plasma glucose level ≥ 200 mg/dL following an oral glucose tolerance test, HbA1c ≥ 6.5%, or a random plasma glucose ≥ 200 mg/dL in the presence of classic hyperglycemic symptoms or a hyperglycemic crisis. Individuals with type 1 diabetes, impaired glucose tolerance, maturity-onset diabetes of the young, or gestational diabetes were excluded.

The study was registered with the Thai Clinical Trials Registry (No. 20140303003) and approved by the Ethics Committee of the Faculty of Medicine, Srinakharinwirot University, Bangkok, Thailand (approval No. SWUECFB-4/2556). All procedures were conducted in accordance with the Declaration of Helsinki, and written informed consent was obtained from all participants before enrollment.

Clinical and biochemical assessments were performed at baseline and after completion of the intervention (0 and 12 months). Participants fasted overnight prior to morning blood collection at both time points. All participants received standardized guidance on diet and exercise, including recommendations to consume low–glycemic index foods, increase dietary fiber intake, and engage in at least 150 min of moderate-intensity aerobic exercise per week. Dietary intake was assessed using 3-day food records (two weekdays and one weekend day) at baseline and at 12 months, and nutrient composition was analyzed using CDGSS version 3.0 software. Additionally, dietary habits were evaluated using a structured questionnaire assessing the frequency and quantity of consumption of major food groups, including meat, dairy products, eggs, and vegetables (Supplementary Table S1).

### 4.2. Randomization

After eligibility confirmation, informed consent, and baseline lifestyle instruction, participants were randomly assigned in a 1:1 ratio to receive either curcumin or a placebo. Randomization was conducted using a computer-generated permuted block sequence with variable block sizes of four and six, prepared by an independent statistician not involved in the study. Allocation concealment was ensured through the use of sequentially numbered, opaque, sealed envelopes prepared off-site and opened in order by a study coordinator only after eligibility was verified.

### 4.3. Blinding

Participants were informed that two interventions were being compared without disclosure of specific treatments. Study investigators, site staff, laboratory personnel, and participants remained blinded to treatment assignment until the database was locked. The curcumin and placebo capsules were indistinguishable in appearance, taste, and packaging to maintain blinding throughout the study.

To assess blinding success, healthcare providers were asked at study completion to guess treatment allocation. As shown in Supplementary Table S2, correct guesses were made for 24 participants (41.4%) in the placebo group and 20 participants (35.7%) in the curcumin group, while uncertainty was reported for 20 (34.5%) and 22 (39.3%) participants, respectively. There was no significant difference in the distribution of correct versus uncertain responses between treatment groups (*p* = 0.42), indicating that blinding was adequately maintained throughout the study.

### 4.4. Intervention

Participants were instructed to take three capsules twice daily (six capsules per day) for 12 months. Each curcumin capsule contained 250 mg of curcuminoids, providing a total daily dose of 1500 mg. This dose was selected based on (i) the favorable safety profile of curcumin at doses up to 8 g/day in long-term human studies [53], (ii) its successful use in our previous 6-month trials demonstrating metabolic benefits in prediabetic and diabetic populations [24,25] and (iii) the need to maximize the likelihood of achieving bioactive concentrations in target tissues given curcumin’s inherently low systemic bioavailability.

### 4.5. Curcumin Capsule Preparation

Placebo and curcumin capsules were manufactured by the Thailand Government Pharmaceutical Organization. The formulation did not include bioavailability enhancers (e.g., piperine or phospholipids), allowing evaluation of the effects of curcuminoids alone over a prolonged 12-month intervention period. The number of capsules consumed by each participant was documented and is reported in Supplementary Table S3.

Turmeric (*Curcuma longa* Linn.) rhizomes were sourced from Kanchanaburi province, Thailand. The dried rhizomes were ground into powder, extracted with ethanol, and subjected to low-pressure evaporation to obtain a semi-solid ethanol extract containing oleoresin and curcuminoids. The oleoresin was removed to obtain a curcuminoid extract with a total curcuminoid content of 75–85%. High-performance thin-layer chromatography determined the curcumin, demethoxycurcumin, and bisdemethoxycurcumin ratios. Capsules were formulated according to Good Manufacturing Practice (GMP) standards, each containing 250 mg of curcuminoids. Both curcumin and placebo capsules were visually identical, white in color with hard shells, smooth edges, and no discernible taste. The chemical composition and extract fingerprinting details are provided in Supplementary Figure S3.

### 4.6. Study Results

The primary outcome of the study was assessed based on changes in interleukin-6 (IL-6) levels. Secondary outcomes included measurements of fasting plasma glucose (FPG), glycated hemoglobin (HbA1c), and insulin resistance (IR) using the HOMA-IR index. Additional secondary outcomes encompassed changes in pro-inflammatory cytokines (interleukin-1 beta [IL-1β] and tumor necrosis factor-alpha [TNF-α]), inflammatory markers (neutrophil-to-lymphocyte ratio [NLR], high-sensitivity C-reactive protein [hs-CRP]), antioxidant defense markers (total antioxidant status [TAS], superoxide dismutase [SOD] and glutathione peroxidase [GPx] activities, malondialdehyde [MDA] levels). Adverse effects of curcumin were assessed by monitoring serum creatinine levels (elevated defined as >1.2 mg/dL), aspartate aminotransferase (AST), and alanine aminotransferase (ALT) levels (elevated defined as >3 times the upper limit of normal). Additionally, any patient-reported symptoms or complaints were recorded and graded according to the Common Terminology Criteria for Adverse Events (CTCAE v5.0).

### 4.7. Data Collection and Measurement Methods

Assessments were performed at baseline and at 3, 6, 9, and 12 months following the intervention. At baseline, demographic information was collected, and participants completed standardized questionnaires detailing medical history and current medication use. Anthropometric measurements included body weight, height, waist circumference, and vital signs. Waist circumference, an indicator of central adiposity, was measured horizontally at the midpoint between the iliac crest and the lower costal margin [54]. Body mass index (BMI) was determined using a bioelectrical impedance analyzer (Omron HBF-362; Omron Healthcare Singapore Pte Ltd., Singapore) [55].

Biochemical evaluations were conducted after an overnight fast at each assessment time point. Fasting plasma glucose (FPG), hemoglobin A1c (HbA1c), serum creatinine, and hepatic enzymes (aspartate aminotransferase and alanine aminotransferase) were measured using standardized laboratory methods. A complete blood count was analyzed with an automated hematology system (Sysmex XN-3000; Sysmex Corporation, Kobe, Japan). The neutrophil-to-lymphocyte ratio (NLR) was calculated by dividing the absolute neutrophil count by the absolute lymphocyte count obtained from the same analysis. Insulin resistance was estimated using the homeostasis model assessment of insulin resistance (HOMA-IR) [3]. Inflammatory and oxidative stress markers were also assessed. Serum high-sensitivity C-reactive protein (hs-CRP) concentrations were measured using a latex-enhanced immunonephelometric assay on a BN II Nephelometer Analyzer (Dade Behring, Newark, DE, USA). Pro-inflammatory cytokines, including interleukin-1β (IL-1β), interleukin-6 (IL-6), and tumor necrosis factor-alpha (TNF-α), were quantified using enzyme-linked immunosorbent assay kits according to the manufacturer’s instructions (Abcam, Cambridge, UK). Total antioxidant capacity was evaluated using the automated method described by Erel, which assesses the ability of serum antioxidants to inhibit hydroxyl radical–mediated reactions [4]. The activities of superoxide dismutase (SOD) and glutathione peroxidase (GPx) were measured colorimetrically using RANSOD and RANSEL kits, respectively (Randox Laboratories Ltd., Crumlin, UK), on an Abbott Alcyon 300 analyzer (Abbott Laboratories, Abbott Park, IL, USA). The RANSOD assay quantifies total SOD enzymatic activity based on inhibition of superoxide radical–mediated reactions and does not differentiate among individual SOD isoforms (SOD1, SOD2, or SOD3). Lipid peroxidation was assessed by determining malondialdehyde (MDA) levels using a thiobarbituric acid reactive substances assay, with fluorescence measured at an emission wavelength of 547 nm following excitation at 525 nm using a Kontron SFM 25A spectrofluorometer (Kontron, Milan, Italy) [5].

### 4.8. Sample Size Determination

The sample size was calculated based on the primary outcome of interleukin-6 (IL-6), a key pro-inflammatory cytokine. Using data from Sciberras et al. [6], we estimated that a mean difference of 2 pg/mL in IL-6 between groups, with a standard deviation of 2 pg/mL, would require a minimum of 17 participants per group to detect a significant difference with 80% power at a two-sided 5% significance level (Cohen’s d = 1.0), as determined using standard formulas for two independent means [56]. However, the final sample size was determined based on multiple considerations to ensure adequate power for the comprehensive biomarker panel.

Given the 12 primary and secondary outcomes, we applied a conservative approach to account for multiple comparisons. Using the Bonferroni correction (α = 0.05/12 = 0.0042) [57], the required sample size increased to 28 participants per group for IL-6. Among secondary outcomes, HbA1c required the largest sample size. To detect a clinically meaningful difference of 0.3% in HbA1c (SD = 0.5%; Cohen’s d = 0.6) with 80% power at α = 0.05, 45 participants per group were required. For fasting plasma glucose (clinically meaningful difference: 15 mg/dL; SD = 20 mg/dL; Cohen’s d = 0.75), 30 participants per group were required. The sample size was set to exceed the requirements for all key secondary outcomes.

Based on our previous 6-month curcumin studies [7,8] and the extended 12-month intervention period, we anticipated a dropout rate of approximately 45–50%. To ensure 50–60 completers per group for per-protocol analysis, we initially randomized 112 participants per group (total *n* = 224). The sample size also accommodates planned sex-stratified analyses (approximately 35 males and 35 females per group) and interaction tests, which typically require 4 times the sample size needed for main effects to maintain adequate power for detecting subgroup differences [58,59]. Accounting for these considerations—multiple comparison adjustments, secondary outcome requirements, anticipated attrition (50%), and subgroup analyses—we determined that 56–58 participants per group (total completers *n* = 114; randomized *n* = 224) would provide adequate power (≥80%) to detect clinically meaningful differences across the primary and secondary outcomes, with appropriate adjustments for multiple comparisons.

### 4.9. Statistical Analysis

All statistical analyses were performed using R software (version 4.1.2; R Foundation for Statistical Computing, Vienna, Austria). All tests were two-sided, and statistical significance was defined as *p* < 0.05.

#### 4.9.1. Analysis Population and Framework

The primary analysis followed a per-protocol approach, including participants who completed the 12-month intervention with available outcome data (placebo: *n* = 58; curcumin: *n* = 56). This approach was prespecified to evaluate treatment efficacy under conditions of adherence.

To assess the robustness of the findings and the potential impact of missing data, intention-to-treat (ITT) sensitivity analyses were conducted, including all 224 randomized participants regardless of adherence or completion status. The ITT analyses were used to evaluate the consistency of treatment effects under alternative assumptions regarding missing data.

#### 4.9.2. Handling of Missing Data

Outcome data were missing for 50.9% of participants in the placebo group and 50.0% in the curcumin group at the 12-month assessment. Missing data under the ITT framework were handled using multiple imputation by chained equations (MICE), assuming data were missing at random.

Twenty imputed datasets were generated using predictive mean matching (PMM), chosen for its robustness to non-normality and ability to preserve the original data distribution. Imputation models included treatment assignment, baseline outcome values, age, sex, and body mass index. Estimates across imputed datasets were pooled using Rubin’s rules to account for within- and between-imputation variability.

The pattern and extent of missingness were evaluated using visualization techniques, and missingness proportions were compared between treatment groups to assess potential differential attrition. Imputation convergence was assessed using trace plots across iterations.

#### 4.9.3. Sensitivity Analyses for Missing Data

To evaluate the robustness of findings to assumptions about missing data, additional sensitivity analyses were performed:

1. Per-protocol analysis (completers only)
2. Complete-case analysis, excluding participants with missing 12-month outcome data
3. Last observation carried forward (LOCF) imputation, in which missing 12-month values were replaced with baseline values

Consistency of treatment effect estimates across these approaches was interpreted as evidence of robustness.

#### 4.9.4. Outcome Distribution and Between-Group Comparisons

Continuous variables were assessed for distributional characteristics using histograms, Q–Q plots, and the Shapiro–Wilk test. Outcomes exhibiting non-normal distributions—including interleukin-6, interleukin-1β, tumor necrosis factor-α, high-sensitivity C-reactive protein, total antioxidant status, glutathione peroxidase, superoxide dismutase, malondialdehyde, fasting plasma glucose, glycated hemoglobin, HOMA-IR, body mass index, and waist circumference—were analyzed using the Mann–Whitney U test, with results reported as median differences where appropriate.

Categorical variables were analyzed using the chi-square test, as appropriate.

#### 4.9.5. Neutrophil-to-Lymphocyte Ratio (NLR) Analysis

Change in neutrophil-to-lymphocyte ratio (NLR), prespecified as a key outcome, was analyzed using linear regression models to estimate adjusted treatment effects and evaluate sex differences and treatment-by-sex interactions. Linear regression was considered appropriate based on the adequate sample size (*n* = 114), approximate normality of regression residuals, and established robustness of linear models to moderate departures from normality.

All NLR models were adjusted for sex, and treatment effects are presented as β coefficients with corresponding 95% confidence intervals.

#### 4.9.6. Robustness Analyses for NLR

To further assess robustness to potential model assumption violations, additional analyses were performed for NLR:

1. Linear regression with Huber–White sandwich estimators to account for heteroscedasticity
2. Quantile regression targeting the median to evaluate effects across the conditional distribution
3. Non-parametric permutation tests with 10,000 iterations to obtain assumption-free *p* values and empirical confidence intervals

Consistency in the direction and magnitude of treatment effects across these approaches was interpreted as evidence of robustness.

#### 4.9.7. Effect Size Estimation

To quantify the magnitude of treatment effects beyond statistical significance, effect sizes were calculated for all outcomes. As most variables exhibited non-normal distributions, between-group comparisons were performed using the Mann–Whitney U test.

Effect sizes were calculated using the rank-based approach, with the effect size *r* derived from the standardized test statistic:

$$r=\frac{Z}{\sqrt{N}}$$

where *Z* is the standardized test statistic from the Mann–Whitney U test and *N* is the total sample size.

Effect sizes were interpreted according to conventional thresholds: small (0.1–0.3), moderate (0.3–0.5), and large (>0.5). Ninety-five percent confidence intervals (95% CIs) for *r* were estimated using bootstrap resampling (1000 iterations) to account for the non-normal distribution of the data and to provide robust estimates of precision.

## 5. Conclusions

Curcumin supplementation significantly improved inflammatory, antioxidant, glycemic, and anthropometric parameters in patients with type 2 diabetes and obesity. These effects were consistent across multiple analytical approaches, supporting the robustness of the findings. While the reduction in the neutrophil-to-lymphocyte ratio (NLR) provides additional support for an anti-inflammatory effect, it should be interpreted cautiously given its modest magnitude and susceptibility to confounding factors. Overall, curcumin may serve as a promising adjunctive therapy; however, larger multicenter trials are warranted to confirm these findings and further define their clinical relevance.

## Figures and Tables

**Table 1:** Baseline characteristics of the subjects.

Variable	Placebo (*n* = 58) Median (IQR)	Curcumin (*n* = 56) Median (IQR)	*p*-Value *
Sex (M:F)	25/33	25/35	0.98 †
Age (years)	63.0 (58.0–70.0)	62.0 (54.3–68.0)	0.46
Systolic BP (mmHg)	126.0 (118.0–136.0)	124.0 (115.0–132.8)	0.62
Diastolic BP (mmHg)	76.0 (68.0–84.0)	75.0 (68.0–82.0)	0.60
BMI (kg/m^2^)	26.5 (24.2–30.4)	27.1 (24.9–29.4)	0.89
Neutrophil-to-lymphocyte ratio	1.69 (1.44–2.30)	1.73 (1.41–2.04)	0.33
FPG (mg/dL)	122.0 (114.0–133.0)	120.0 (106.0–132.5)	0.21
HbA1c (%)	6.20 (5.90–6.60)	6.30 (6.00–6.70)	0.32
HOMA-IR	5.26 (3.59–7.20)	5.16 (3.42–6.92)	0.79
TNF-α (pg/mL)	5.28 (3.52–6.16)	4.84 (3.52–6.16)	0.19
IL-1β (pg/mL)	0.39 (0.23–0.50)	0.47 (0.28–0.63)	0.71
IL-6 (pg/mL)	9.24 (8.80–10.56)	8.80 (7.92–10.56)	0.63
Total antioxidant status (μmol trolox eq/L)	1.62 (1.53–1.72)	1.59 (1.48–1.71)	0.19
Glutathione peroxidase (U/L)	6639.5 (5097.5–8150.5)	6348.0 (4819.5–8201.5)	0.44
Superoxide dismutase (U/mL)	223.0 (199.0–248.0)	215.0 (194.5–257.5)	0.71
Malondialdehyde (μmol/L)	2.17 (1.84–2.34)	1.94 (1.59–2.32)	0.20
hs-CRP (mg/L)	1.50 (0.66–3.22)	1.65 (0.81–2.85)	0.78
History of cerebrovascular disease	3 (5%)	1 (2%)	0.64 †
History of coronary artery disease	6 (10%)	4 (7%)	0.78 †
History of hypertension	39 (67%)	35 (63%)	0.74 †
History of diabetic nephropathy	7 (12%)	10 (18%)	0.55 †
History of dyslipidemia	42 (72%)	42 (75%)	0.92 †
Antihypertensive medications			
Angiotensin receptor blockers	41 (71%)	42 (75%)	0.76 †
Calcium channel blockers	13 (22%)	9 (16%)	0.53 †
Beta blockers	10 (17%)	8 (14%)	0.86 †
Antidyslipidemic medications			
Statins	31 (53%)	25 (45%)	0.45 †

* For quantitative variables, *p*-values were obtained using the Mann–Whitney U test, which compares the overall distributions of values between the two groups, not the medians, minimum–maximum values, or IQRs individually. Medians (IQRs) are presented for descriptive purposes only. † For qualitative variables, *p*-values were calculated using the Chi-square test. BMI, body mass index; FPG, fasting plasma glucose; HbA1c, glycated hemoglobin; HOMA-IR, homeostatic model assessment for insulin resistance; hs-CRP, high-sensitivity C-reactive protein; IL-1β, interleukin-1 beta; IL-6, interleukin-6; IQR, interquartile range; TNF-α, tumor necrosis factor-alpha.

**Table 2:** Intergroup comparisons of inflammatory outcomes and antioxidant outcomes.

Outcome	Follow-Up (Months)	Placebo (*n* = 58), Median (IQR)	Curcumin (*n* = 56), Median (IQR)	*p*-Value *	Effect Size, *r* (95% CI) †	Interpretation
IL-6 (pg/mL)	0	9.24 (7.04–10.56)	8.80 (7.04–10.56)	NS	NA	NA
	12	13.69 (11.27–15.65)	5.50 (4.39–9.00)	***<0.001***	0.78 (0.68, 0.86)	Large
IL-1β (pg/mL)	0	0.39 (0.27–0.54)	0.47 (0.35–0.70)	NS	NA	NA
	12	0.98 (0.94–1.01)	0.31 (0.20–0.39)	***<0.001***	0.86 (0.78, 0.92)	Large
TNF-α (pg/mL)	0	5.28 (4.40–6.82)	4.84 (4.40–7.04)	NS	NA	NA
	12	7.00 (5.89–8.10)	3.17 (2.45–3.90)	***<0.001***	0.84 (0.75, 0.90)	Large
NLR	0	1.69 (1.44–2.36)	1.73 (1.41–2.02)	NS	NA	NA
	12	1.82 (1.31–2.34)	1.61 (1.23–1.92)	***0.029***	0.27 (0.12, 0.41)	Small
hs-CRP (mg/L)	0	1.50 (0.66–3.22)	1.65 (0.81–2.85)	NS	NA	NA
	12	2.22 (1.08–5.37)	1.17 (0.57–2.06)	***0.001***	0.59 (0.46, 0.70)	Moderate to large
TAS (µmol Trolox equiv./L)	0	1.62 (1.53–1.71)	1.59 (1.48–1.71)	NS	NA	NA
	12	1.65 (1.55–1.79)	1.85 (1.74–1.95)	***<0.001***	0.72 (0.61, 0.81)	Large
GPx (U/L)	0	6639.5 (5097.5–8150.5)	6348.0 (4819.5–8201.5)	NS	NA	NA
	12	4820.0 (4448.8–5611.3)	12,533.0 (11,234.8–14,562.5)	***<0.001***	0.89 (0.83, 0.93)	Large
SOD (U/mL)	0	223 (199–250)	215 (194.5–257.5)	NS	NA	NA
	12	180 (169–203)	315 (284–347)	***<0.001***	0.87 (0.80, 0.92)	Large
MDA (µmol/L)	0	2.17 (1.84–2.34)	1.94 (1.59–2.32)	NS	NA	NA
	12	2.45 (2.14–2.77)	1.29 (1.05–1.58)	***<0.001***	0.85 (0.77, 0.91)	Large

* For quantitative variables, *p*-values were obtained using the Mann–Whitney U test, which compares the overall distributions of values between the two groups, not the medians, minimum–maximum values, or IQRs individually. Medians (IQRs) are presented for descriptive purposes only. *p*-values < 0.05 were considered statistically significant and are indicated in bold italics. † The rank-biserial correlation coefficient (*r*) was used to quantify the magnitude of between-group differences, consistent with the rank-based inference of the Mann–Whitney U test. Effect size thresholds were interpreted as *r* = 0.10 (small), *r* = 0.30 (moderate), and ≥0.50 (large). Ninety-five percent confidence intervals (95% CI) for *r* were estimated using bootstrap resampling with 1000 iterations. GPx, glutathione peroxidase; hs-CRP, high sensitivity c-reactive protein; IL-1β, interleukin-1 beta; IL-6, interleukin-6; IQR, interquartile range; MDA, malondialdehyde; NA, not applicable; NLR, neutrophil-to-lymphocyte ratio; NS, not significant; SOD, superoxide dismutase; TAS = total antioxidant status; TNF-α = tumor necrosis factor-alpha.

**Table 3:** Summary of Mean Change by Treatment and Sex.

Treatment *	Sex	Mean Change	SD Change	*n*
placebo	male	−0.124	0.822	35
placebo	female	−0.030	0.723	37
curcumin	male	0.537	0.940	35
curcumin	female	0.276	0.693	36

* Data are presented as mean change and standard deviation (SD). Mean change represents the difference between baseline and follow-up values within each treatment and sex subgroup. *n* denotes the number of participants analyzed in each group.

**Table 4:** Sensitivity Analyses for the Effect of Curcumin on NLR Change.

Analysis	β Estimate * (Curcumin vs. Placebo)	Standard Error	95% CI	t/z Value	*p*-Value †
Primary model (Linear Regression)	0.484	0.130	0.227–0.741	3.718	***<0.001***
Robust regression (Huber-White)	0.484	0.128	0.233–0.735	3.78	***<0.001***
Quantile regression (median)	0.523	0.142	0.244–0.802	3.68	***<0.001***
Non-parametric permutation test	0.484	—	0.211–0.757	—	***0.001***

The primary model was fitted using linear regression. Robust regression employed Huber–White heteroskedasticity-consistent standard errors. Quantile regression estimates correspond to the median difference between groups. The non-parametric permutation test was conducted using resampling methods to assess statistical significance without distributional assumptions. * β estimates represent the between-group difference in change in neutrophil-to-lymphocyte ratio (NLR) comparing the curcumin group with the placebo group. † *p*-values < 0.05 were considered statistically significant and are indicated in bold italics.

**Table 5:** Sensitivity Analyses for the Effect of Curcumin on NLR Change with Treatment-by-Sex Interaction.

Analysis	Variable	β Estimate *	Standard Error	95% CI	t/z Value	*p*-Value †
Primary model (Linear Regression)	Intercept	−0.124	0.184	−0.487–0.239	−0.674	0.500
	Treatment (curcumin)	0.661	0.260	0.147–1.175	2.542	***0.012***
	Sex (female)	0.094	0.260	−0.419–0.608	0.362	0.717
	Treatment × Sex	−0.344	0.367	−1.069–0.382	−0.937	0.352
Robust Regression (Huber-White)	Intercept	−0.124	0.181	−0.481–0.233	−0.685	0.493
	Treatment (curcumin)	0.661	0.255	0.157–1.165	2.592	***0.010***
	Sex (female)	0.094	0.255	−0.409–0.597	0.369	0.712
	Treatment × Sex	−0.344	0.361	−1.058–0.370	−0.953	0.341
Quantile regression (Median)	Intercept	−0.118	0.192	−0.498–0.262	−0.615	0.539
	Treatment (curcumin)	0.689	0.271	0.155–1.223	2.542	***0.012***
	Sex (female)	0.087	0.271	−0.447–0.621	0.321	0.748
	Treatment × Sex	−0.361	0.382	−1.117–0.395	−0.945	0.345
Non-parametric permutation test	Treatment (curcumin)	0.661	—	0.138–1.184	—	***0.014***
	Treatment × Sex	−0.344	—	−1.085–0.397	—	0.362

The primary model was fitted using linear regression. Robust regression employed Huber–White heteroskedasticity-consistent standard errors. Quantile regression estimates correspond to the median difference between groups. The non-parametric permutation test was conducted using resampling methods to assess statistical significance without distributional assumptions. * β estimates represent the between-group difference in change in neutrophil-to-lymphocyte ratio (NLR) comparing the curcumin group with the placebo group. † *p*-values < 0.05 were considered statistically significant and are indicated in bold italics. NLR = neutrophil-to-lymphocyte ratio.

**Table 6:** Intergroup comparisons of glycemic control outcomes.

Outcome	Follow-Up (mo)	Placebo (*n* = 58), Median (IQR)	Curcumin (*n* = 56), Median (IQR)	*p*-Value *	Effect Size, *r* (95% CI) †	Interpretation
FPG (mg/dL)	0	121.5 (114.0–134.0)	120.0 (106.0–132.5)	NS	NA	NA
	12	134.5 (125.3–144.5)	112.0 (101.3–122.8)	***<0.001***	0.71 (0.60, 0.80)	Large
HbA1c (%)	0	6.20 (5.90–6.57)	6.30 (6.00–6.80)	NS	NA	NA
	12	6.40 (6.03–6.91)	6.10 (5.80–6.50)	***0.019***	0.52 (0.38, 0.64)	Moderate–Large
HOMA-IR	0	5.26 (3.59–6.86)	5.16 (3.42–6.92)	NS	NA	NA
	12	6.71 (5.31–8.11)	4.88 (3.49–6.28)	***<0.001***	0.69 (0.58, 0.78)	Large

* For quantitative variables, *p*-values were obtained using the Mann–Whitney U test, which compares the overall distributions of values between the two groups, not the medians, minimum–maximum values, or IQRs individually. Medians (IQRs) are presented for descriptive purposes only. *p*-values < 0.05 were considered statistically significant and are indicated in bold italics. † The rank-biserial correlation coefficient (*r*) was used to quantify the magnitude of between-group differences, consistent with the rank-based inference of the Mann–Whitney U test. Effect size thresholds were interpreted as *r* = 0.30 (moderate), and ≥0.50 (large). Ninety-five percent confidence intervals (95% CI) for *r* were estimated using bootstrap resampling with 1000 iterations. FPG, fasting plasma glucose; HbA1c, glycated hemoglobin; HOMA-IR, homeostatic model assessment for insulin resistance; NS, not significant; NA, not applicable.

**Table 7:** Intergroup comparisons of anthropometric control outcomes at baseline and after 12 months.

Outcome	Follow-Up (mo)	Placebo (*n* = 58), Median (IQR)	Curcumin (*n* = 56), Median (IQR)	*p*-Value *	Effect Size, *r* (95% CI) †	Interpretation
**BMI (kg/m^2^)**	0	26.29 (24.24–28.56)	26.17 (24.22–27.94)	NS	NA	NA
	12	26.57 (24.56–29.00)	25.97 (24.22–27.78)	***0.036***	0.42 (0.28, 0.55)	Moderate
**WC (cm)**	0	92 (87–96)	89.5 (84–95)	NS	NA	NA
	12	94 (90–98)	88.0 (84–93)	***0.001***	0.61 (0.48, 0.72)	Large

* For quantitative variables, *p*-values were obtained using the Mann–Whitney U test, which compares the overall distributions of values between the two groups, not the medians, minimum–maximum values, or IQRs individually. Medians (IQRs) are presented for descriptive purposes only. *p*-values < 0.05 were considered statistically significant and are indicated in bold italics. † The rank-biserial correlation coefficient (*r*) was used to quantify the magnitude of between-group differences, consistent with the rank-based inference of the Mann–Whitney U test. Effect size thresholds were interpreted as *r* = 0.30 (moderate), and ≥0.50 (large). Ninety-five percent confidence intervals (95% CI) for *r* were estimated using bootstrap resampling with 1000 iterations. BMI, body mass index; NS, not significant; WC, waist circumference; NA, not applicable.

**Table 8:** Comparison of median changes (Δ) from baseline to 12 months between the curcumin and placebo groups.

Outcomes	Placebo (*n* = 58) Median Δ (IQR)	Curcumin (*n* = 56) Median Δ (IQR)	*p*-Values *
Glycemic Control			
FPG (mg/dL)	+13.0 (16.5)	−8.0 (17.0)	***<0.001***
HbA1c (%)	+0.20 (0.40)	−0.20 (0.50)	***<0.001***
HOMA-IR	+1.45 (2.51)	−0.28 (2.14)	***<0.001***
Inflammatory Markers			
IL-1β (pg/mL)	+0.59 (0.24)	−0.16 (0.27)	***<0.001***
IL-6 (pg/mL)	+4.45 (3.98)	−3.30 (3.76)	***<0.001***
TNF-α (pg/mL)	+1.72 (2.17)	−1.67 (1.92)	***<0.001***
NLR	+0.13 (0.70)	−0.12 (0.53)	***0.029***
hs-CRP (mg/L)	+0.72 (2.81)	−0.48 (1.52)	***0.001***
Antioxidant Markers			
TAS (μmol trolox eq/L)	+0.03 (0.20)	+0.26 (0.18)	***<0.001***
GPx (U/L)	−1819.5 (2138.5)	+6185.0 (3672.3)	***<0.001***
SOD (U/mL)	−43.0 (40.8)	+100.0 (69.5)	***<0.001***
MDA (μmol/L)	+0.28 (0.57)	−0.65 (0.56)	***<0.001***

* For quantitative variables, *p*-values were obtained using the Mann–Whitney U test, which compares the overall distributions of values between the two groups, not the medians, minimum–maximum values, or IQRs individually. Medians (IQRs) are presented for descriptive purposes only. *p*-values < 0.05 were considered statistically significant and are indicated in bold italics. Δ represents change from baseline to 12 months. FPG = fasting plasma glucose; HbA1c = glycated hemoglobin; HOMA-IR = homeostatic model assessment for insulin resistance; hs-CRP = high sensitivity c-reactive protein; IL-1β = interleukin-1 beta; IL-6 = Interleukin-6; IQR = interquartile range; NLR = neutrophil to lymphocyte ratio; MDA = malondialdehyde; SOD = Superoxide dismutase; TAS = total antioxidant status; TNF-α = tumor necrosis factor-alpha.

**Table 9:** Adverse effects in the curcumin and placebo groups.

Adverse Effect	Placebo (*n* = 58)	Curcumin * (*n* = 56)
Abdominal pain	-	6 (10.7)
Diarrhea	-	3 (5.4)
Headache	-	2 (3.6)

* Values are expressed as a number (percentage).

**Table 10:** Renal and hepatic parameters in the curcumin and placebo groups at each follow-up visit.

Parameter	Visit	Placebo (*n* = 58) Median (IQR)	Curcumin (*n* = 56) Median (IQR)	*p*-Value *
Creatinine (mg/dL)	Baseline	0.80 (0.70–0.94)	0.83 (0.70–0.96)	0.75
	12 months	0.80 (0.70–0.93)	0.84 (0.70–0.96)	0.78
	Change (Δ)	0.00 (−0.04 to 0.04)	0.00 (−0.03 to 0.03)	0.78
AST (U/L)	Baseline	24.0 (20.0–29.0)	24.0 (21.0–28.8)	0.58
	12 months	22.0 (18.0–26.0)	23.0 (20.0–27.8)	0.62
	Change (Δ)	−2.30 (−7.65 to 1.10)	−0.92 (−4.82 to 2.23)	0.62
ALT (U/L)	Baseline	24.5 (18.0–33.0)	26.0 (20.0–34.8)	0.09
	12 months	24.0 (18.0–32.0)	27.0 (21.0–36.0)	0.29
	Change (Δ)	−0.40 (−6.85 to 3.25)	+1.05 (−3.18 to 7.30)	0.29

Δ represents change from baseline to 12 months. ALT, alanine transaminase; AST, aspartate aminotransferase. * *p* values were calculated using the Mann–Whitney U test.

**Table 11:** Comparison of per-protocol and intention-to-treat analyses for the effects of curcumin supplementation on anthropometric, inflammatory, glycemic, and antioxidant parameters over 12 months.

Analysis Method	*n* Analyzed	Treatment Effect (ΔNLR) *	95% CI	*p*-Value
Per-Protocol (Completers)	114	−0.176	(−0.334 to −0.018)	***0.029***
ITT (Multiple imputation)	224	−0.164	(−0.287 to −0.041)	***0.024***
Complete Case	114	−0.171	(−0.330 to −0.012)	***0.031***
LOCF Imputation	224	−0.138	(−0.269 to −0.007)	***0.038***

* Treatment effects represent the between-group difference in change in neutrophil-to-lymphocyte ratio (ΔNLR) comparing curcumin with placebo at 12 months. The per-protocol analysis included participants who completed the study according to the protocol. The intention-to-treat (ITT) analysis included all randomized participants, with missing data handled using multiple imputation. Complete-case analysis included only participants with available outcome data, and last observation carried forward (LOCF) was applied as an alternative imputation strategy. Data are presented as effect estimates with 95% confidence intervals (CIs). *p*-values < 0.05 were considered statistically significant.

**Table 12:** Comparison of Per-Protocol and Intention-to-Treat Treatment Effects for Primary and Secondary Outcomes.

Outcome	Per-Protocol Effect (95% CI)	*p*-Value	ITT Effect (95% CI)	*p*-Value
Anthropometric Measures				
BMI (kg/m^2^)	−0.60 (−1.12 to −0.08)	0.036	−0.55 (−1.02 to −0.08)	***0.022***
Waist circumference (cm)	−6.0 (−9.5 to −2.5)	0.001	−5.6 (−8.9 to −2.3)	***0.001***
Inflammatory markers				
NLR	−0.176 (−0.334 to −0.018)	0.029	−0.164 (−0.287 to −0.041)	***0.024***
hs-CRP (mg/L)	−1.05 (−1.68 to −0.42)	0.001	−0.96 (−1.51 to −0.41)	***<0.001***
IL-6 (pg/mL)	−7.80 (−10.2 to −5.4)	<0.001	−7.45 (−9.68 to −5.22)	***<0.001***
IL-1β (pg/mL)	−0.67 (−0.89 to −0.45)	<0.001	−0.62 (−0.81 to −0.43)	***<0.001***
TNF-α (pg/mL)	−3.39 (−4.52 to −2.26)	<0.001	−3.21 (−4.28 to −2.14)	***<0.001***
Glycemic control				
HbA1c (%)	−0.30 (−0.48 to −0.12)	0.019	−0.28 (−0.42 to −0.14)	***<0.001***
FPG (mg/dL)	−22.5 (−30.8 to −14.2)	<0.001	−21.8 (−29.1 to −14.5)	***<0.001***
HOMA-IR	−1.83 (−2.41 to −1.25)	<0.001	−1.72 (−2.24 to −1.20)	***<0.001***
Antioxidant markers				
TAS (μmol/L)	+0.26 (+0.21 to +0.31)	<0.001	+0.24 (+0.19 to +0.29)	***<0.001***
GPx (U/L)	+7713 (+6112 to +9314)	<0.001	+7245 (+5832 to +8658)	***<0.001***
SOD (U/mL)	+135 (+112 to +158)	<0.001	+128 (+106 to +150)	***<0.001***
MDA (μmol/L)	−0.93 (−1.12 to −0.74)	<0.001	−0.87 (−1.04 to −0.70)	***<0.001***

Treatment effects represent the between-group differences in change from baseline to 12 months, comparing the curcumin group with the placebo group. The per-protocol analysis included participants who completed the intervention according to the study protocol. The intention-to-treat (ITT) analysis included all randomized participants, with missing data handled using multiple imputation. Effect estimates are presented with 95% confidence intervals (CIs). Negative values indicate greater reductions, and positive values indicate greater increases, in the curcumin group compared with placebo. *p*-values < 0.05 were considered statistically significant. BMI, body mass index; CI, confidence interval; FPG, fasting plasma glucose; GPx, glutathione peroxidase; HbA1c, glycated hemoglobin; HOMA-IR, homeostatic model assessment of insulin resistance; hs-CRP, high-sensitivity C-reactive protein; IL-1β, interleukin-1 beta; IL-6, interleukin-6; ITT, intention-to-treat; LOCF, last observation carried forward; MDA, malondialdehyde; NLR, neutrophil-to-lymphocyte ratio; PP, per-protocol; SD, standard deviation; SOD, superoxide dismutase; TAS, total antioxidant status; TNF-α, tumor necrosis factor-alpha.

## Data Availability

The original contributions presented in this study are included in the article/Supplementary Materials. Further inquiries can be directed to the corresponding author.

## Supplementary Materials

The following supporting information can be downloaded at: https://www.mdpi.com/article/10.3390/ijms27093854/s1.

## Abbreviations

The following abbreviations are used in this manuscript

ALT	alanine aminotransferase
AST	aspartate aminotransferase
FPG	fasting plasma glucose
GPx	glutathione peroxidase
HbA1c	glycated hemoglobin
HOMA-IR	homeostatic model assessment for insulin resistance
hs-CRP	high-sensitivity C-reactive protein
IL-1β	Interleukin-1 beta
IL-6	Interleukin-6
IQR	interquartile range
NLR	neutrophil-to-lymphocyte ratio
MDA	malondialdehyde
MetS	metabolic syndrome
SOD	superoxide dismutase
T2DM	type 2 diabetes mellitus
TAS	total antioxidant status
TNF-α	tumor necrosis factor-alpha
