# Supplementary material for: Curcumin Supplementation Reduces Inflammation, Neutrophil-to-Lymphocyte Ratio (NLR), and Antioxidant Status in Obese Patients with Type 2 Diabetes: A Randomized Controlled Trial
Source: Int J Mol Sci. 2026 Apr 27;27(9):3854. doi: 10.3390/ijms27093854 (PMC13163827; doi:10.3390/ijms27093854)
Supplement: Supplementary file 1 [file ijms-27-03854-s001.zip › ijms-4212016-supplementary.pdf]

Supplementary material

Supplementary Figure S1 CONSORT 2010 checklist: required information for reporting a randomized trial

From: CONSORT 2010 Statement: updated guidelines for reporting parallel group randomized trials

CONSORT 2010 checklist of information to include when reporting a randomised trial*

Section/Topic	Item No	Checklist item	Reported on page No
**Title and abstract**			
	1a	Identification as a randomised trial in the title	1
	1b	Structured summary of trial design, methods, results, and conclusions (for specific guidance see CONSORT for abstracts)	1-2
**Introduction**			
Background and objectives	2a	Scientific background and explanation of rationale	2-4
	2b	Specific objectives or hypotheses	4
**Methods**			
Trial design	3a	Description of trial design (such as parallel, factorial) including allocation ratio	30
	3b	Important changes to methods after trial commencement (such as eligibility criteria), with reasons	
Participants	4a	Eligibility criteria for participants	29
	4b	Settings and locations where the data were collected	29

Interventions	5	The interventions for each group with sufficient details to allow replication, including how and when they were actually administered	31
Outcomes	6a	Completely defined pre-specified primary and secondary outcome measures, including how and when they were assessed	32
	6b	Any changes to trial outcomes after the trial commenced, with reasons	
Sample size	7a	How sample size was determined	30
	7b	When applicable, explanation of any interim analyses and stopping guidelines	
Randomisation:			
Sequence generation	8a	Method used to generate the random allocation sequence	30
	8b	Type of randomisation; details of any restriction (such as blocking and block size)	30
Allocation concealment mechanism	9	Mechanism used to implement the random allocation sequence (such as sequentially numbered containers), describing any steps taken to conceal the sequence until interventions were assigned	30
Implementation	10	Who generated the random allocation sequence, who enrolled participants, and who assigned participants to interventions	
Blinding	11a	If done, who was blinded after assignment to interventions (for example, participants, care providers, those assessing outcomes) and how	30-31
	11b	If relevant, description of the similarity of interventions	
Statistical methods	12a	Statistical methods used to compare groups for primary and secondary outcomes	34
	12b	Methods for additional analyses, such as subgroup analyses and adjusted analyses	
**Results**			
	13a	For each group, the numbers of participants who were randomly assigned, received intended treatment, and were analysed for the primary outcome	

Participant flow (a diagram is strongly recommended)	13b	For each group, losses and exclusions after randomisation, together with reasons	5
Recruitment	14a	Dates defining the periods of recruitment and follow-up	
	14b	Why the trial ended or was stopped	7
Baseline data	15	A table showing baseline demographic and clinical characteristics for each group	6
Numbers analysed	16	For each group, number of participants (denominator) included in each analysis and whether the analysis was by original assigned groups	
Outcomes and estimation	17a	For each primary and secondary outcome, results for each group, and the estimated effect size and its precision (such as 95% confidence interval)	9
	17b	For binary outcomes, presentation of both absolute and relative effect sizes is recommended	
Ancillary analyses	18	Results of any other analyses performed, including subgroup analyses and adjusted analyses, distinguishing pre-specified from exploratory	
Harms	19	All important harms or unintended effects in each group (for specific guidance see CONSORT for harms)	11-12
**Discussion**			
Limitations	20	Trial limitations, addressing sources of potential bias, imprecision, and, if relevant, multiplicity of analyses	27-28
Generalisability	21	Generalisability (external validity, applicability) of the trial findings	15-16
Interpretation	22	Interpretation consistent with results, balancing benefits and harms, and considering other relevant evidence	5-24
**Other information**			
Registration	23	Registration number and name of trial registry	38
Protocol	24	Where the full trial protocol can be accessed, if available	
Funding	25	Sources of funding and other support (such as supply of drugs), role of funders	

Citation: Schulz KF, Altman DG, Moher D, for the CONSORT Group. CONSORT 2010 Statement: updated guidelines for reporting parallel group randomised trials. BMC Medicine. 2010;8:18.
2010 Schulz et al. This is an Open Access article distributed under the terms of the Creative Commons Attribution License (http://creativecommons.org/licenses/by/2.0), which permits unrestricted use, distribution, and reproduction in any medium, provided the original work is properly cited.

*We strongly recommend reading this statement in conjunction with the CONSORT 2010 Explanation and Elaboration for important clarifications on all the items. If relevant, we also recommend reading CONSORT extensions for cluster randomised trials, non-inferiority and equivalence trials, non-pharmacological treatments, herbal interventions, and pragmatic trials. Additional extensions are forthcoming: for those and for up-to-date references relevant to this checklist, see www.consort-statement.org.

Supplementary Figure S2. Flowchart of the study protocol.

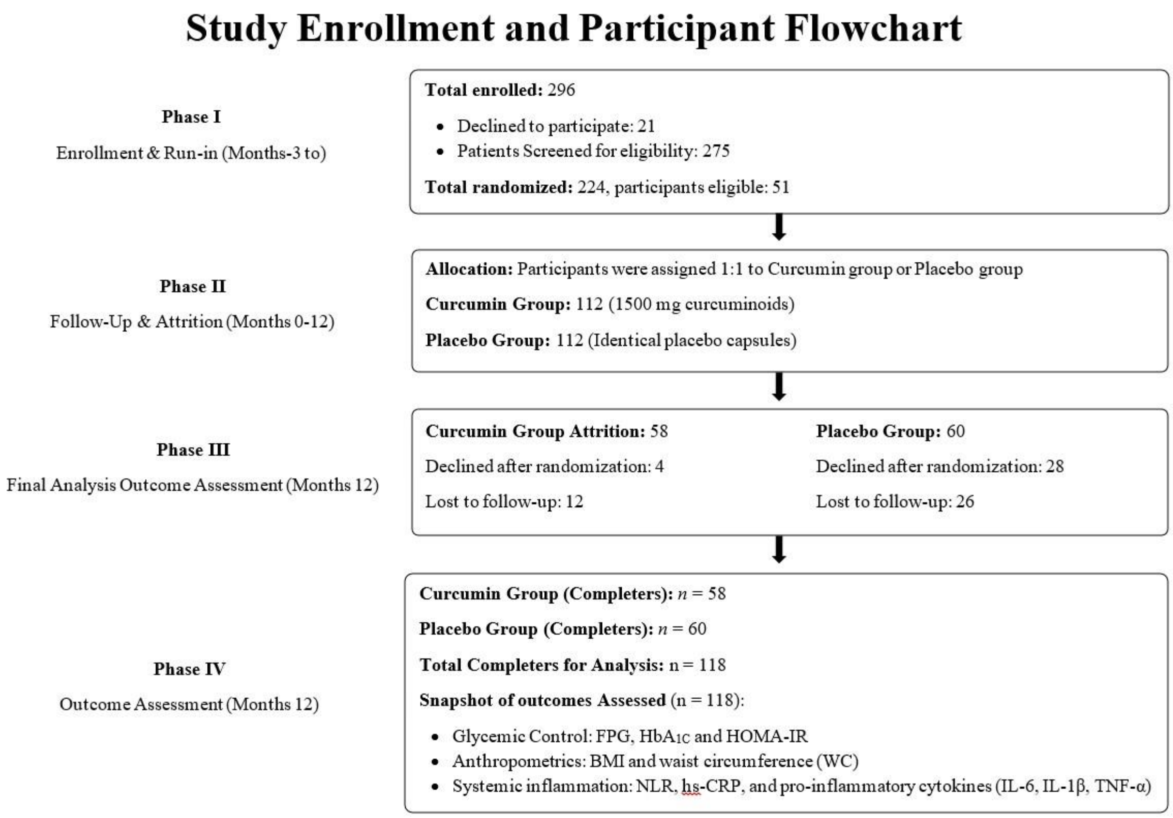

The participant flow is shown in Figure S2. Of 296 individuals screened, eligible participants completed a 3-month lifestyle run-in followed by baseline assessment and 1:1 randomization to curcumin or placebo under double-blind conditions. Participants received the assigned intervention for 12 months in addition to metformin monotherapy, after which final outcome assessments were performed and compared with baseline values.

Abbreviations: BMI, body mass index; FPG, fasting plasma glucose; HbA1c, glycated hemoglobin; HOMA-IR, homeostasis model assessment of insulin resistance; NLR, neutrophil-to-lymphocyte ratio; hs-CRP, high-sensitivity C-reactive protein; IL-6, interleukin-6; IL-1β, interleukin-1 beta; TNF-α, tumor necrosis factor alpha; TAS, total antioxidant status; SOD, superoxide dismutase; GPx, glutathione peroxidase; MDA, malondialdehyde.

Supplementary Figure S3 High-performance thin-layer chromatography profiles of curcuminoid extracts compared to standard curcuminoid chromatograms

The high-performance thin-layer chromatography (HPTLC) chromatogram of the Thai Government Pharmaceutical Organization (GPO) extracts is shown in fig 2, compared with HPTLC chromatogram of standard across curcumin (curcuminoids) in fig 1. In every batch of GPO curcuminoids extract, the peak ratio of curcumin to demethoxycurcumin to bisdemethoxycurcumin was controlled to be 1 to not more than 0.6 to not more than 0.4.

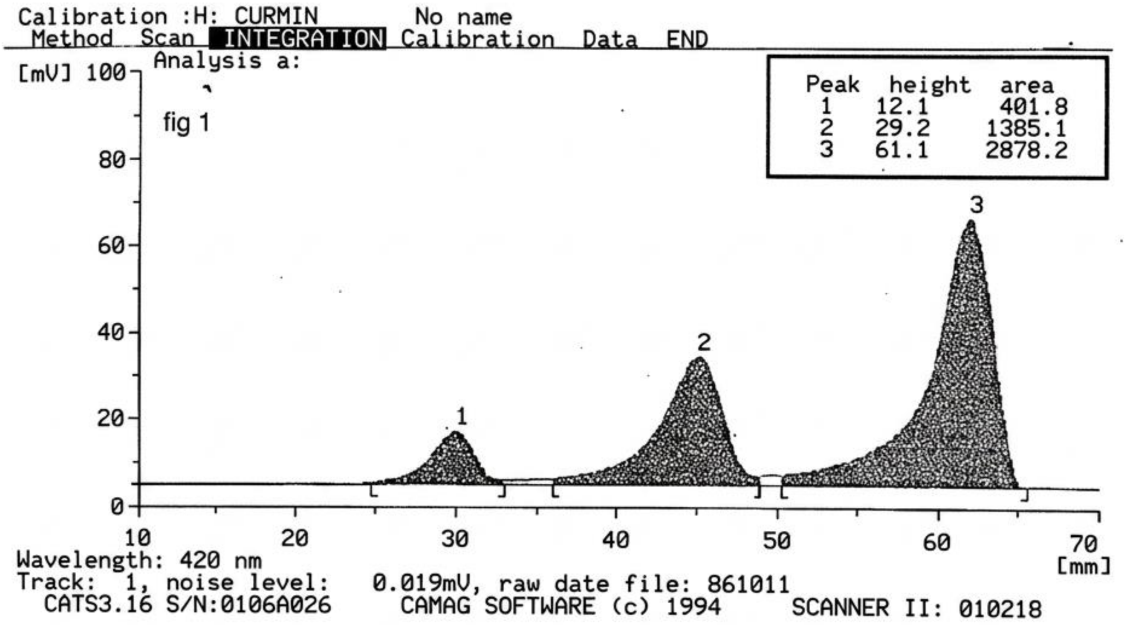

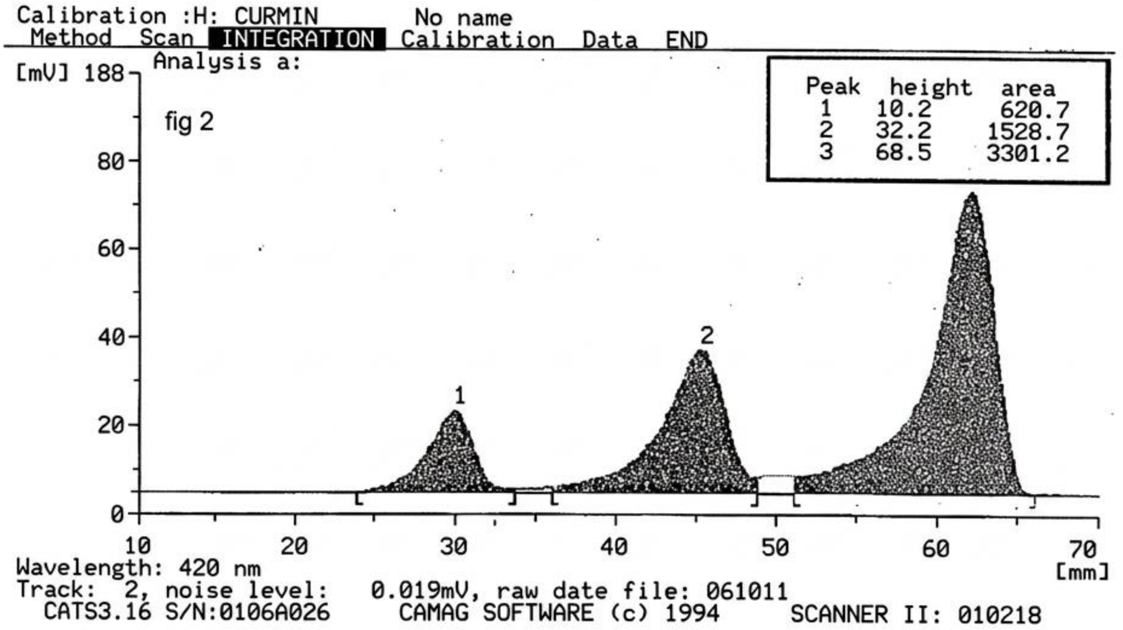

Supplementary Table S1 Mean daily nutrient intake at baseline and 12 months, by treatment group

Daily intake of nutrients	Placebo (*n*=58)		Curcumin (*n*=56)		*P* value [2]
	Baseline[1]	12 months	Baseline[1]	12 months	
Energy (kcal/d)	1897.60±90.98	1913.74±87.25	1915.21±87.98	1908.16±79.53	0.110
Carbohydrate (% of energy)	57.50±2.57	58.07±2.56	57.04±1.52	58.08±1.62	0.148
Protein (% of energy)	12.98±2.13	13.21±1.35	13.33±1.28	13.44±1.20	0.418
FAT (% of energy)	28.27±2.12	28.91±1.91	28.47±2.42	28.44±2.23	0.056
Fiber (g/d)	8.54±1.16	8.46±0.88	8.49±0.82	8.39±0.64	0.464

[1] All parameters are presented as means ± SDs. There are no significant differences between two groups at baseline for any variable by *t* test.

[2] The curcumin had no significant effect on mean daily intake of nutrients by one-factor ANCOVA with the baseline value as the covariate.

There were no significant differences in the daily mean-energy (energy, carbohydrate, protein, fat, and fiber) and nutrient intakes between the curcumin and placebo groups.

Supplementary Table S2 Blinding assessment outcomes in the placebo and curcumin groups based on healthcare provider guesses.

Parameter	Placebo	Curcumin	Overall
Correct guesses, *n* (%)	24 (41.4%)	20 (35.7%)	44 (38.6%)
Uncertain, *n* (%)	20 (34.5%)	22 (39.3%)	42 (36.8%)
Chi-square *p*-value*	0.42		

Blinding assessment was conducted at study completion by asking healthcare providers to guess treatment allocation (placebo, curcumin, or uncertain) for each participant.

* Chi-square test comparing correct guesses versus uncertain responses between placebo and curcumin groups ($p > 0.05$, not significant).

Supplementary Table S3 Capsule consumption per participant over 3-month intervals and daily intake

**Consumption per 6 months**	**Months**	**Placebo (*n*=58)**	**Curcumin (*n*=56)**	***P* value**
		**Number of capsules taken (mean, SE)**	**Number of capsules taken (mean, SE)**	
	6 months	1069.64 (20.58)	1073.25 (21.14)	0.43
**Consumption per day**	12months	1070.36 (20.34)	1071.84 (21.27)	0.33
	6 months	5.94 (0.23)	5.96 (0.24)	0.42
	12 months	5.95 (0.22)	5.95 (0.24)	0.31
